# Sensing Glucose in the Central Melanocortin Circuits of Rainbow Trout: A Morphological Study

**DOI:** 10.3389/fendo.2019.00254

**Published:** 2019-04-18

**Authors:** Cristina Otero-Rodiño, Ana Rocha, Elisa Sánchez, Rosa Álvarez-Otero, José L. Soengas, José M. Cerdá-Reverter

**Affiliations:** ^1^Laboratorio de Fisioloxía Animal, Departamento de Bioloxía Funcional e Ciencias da Saúde, Facultade de Bioloxía and Centro de Investigación Mariña, Universidade de Vigo, Vigo, Spain; ^2^Grupo Control de Ingesta, Consejo Superior de Investigaciones Científicas (IATS-CSIC), Departamento de Fisiología y Biotecnología de Peces, Instituto de Acuicultura de Torre de la Sal, Castellón, Spain

**Keywords:** glucosensing, glucokinase (GK), AGRP, POMC, neuron, brain, fish

## Abstract

In mammals, glucosensing markers reside in brain areas known to play an important role in the control of food intake. The best characterized glucosensing mechanism is that dependent on glucokinase (GK) whose activation by increased levels of glucose leads in specific hypothalamic neurons to decreased or increased activity, ultimately leading to decreased food intake. In fish, evidence obtained in recent years suggested the presence of GK-like immunoreactive cells in different brain areas related to food intake control. However, it has not been established yet whether or not those neuronal populations having glucosensing capacity are the same that express the neuropeptides involved in the metabolic control of food intake. Therefore, we assessed through dual fluorescent *in situ* hybridization the possible expression of GK in the melanocortinergic neurons expressing proopiomelanocortin (POMC) or agouti-related protein (AGRP). POMC and AGRP expression localized exclusively in the rostral hypothalamus, in the ventral pole of the lateral tuberal nucleus, the homolog of the mammalian arcuate nucleus. Hypothalamic GK expression confined to the ependymal cells coating the ventral pole of the third ventricle but some expression level occurred in the AGRP neurons. GK expression seems to be absent in the hypothalamic POMC neurons. These results suggest that AGRP neurons might sense glucose directly through a mechanism involving GK. In contrast, POMC neurons would not directly respond to glucose through GK and would require presynaptic inputs to sense glucose. Ependymal cells could play a critical role relying glucose metabolic information to the central circuitry regulating food intake in fish, especially in POMC neurons.

## Introduction

Animals must know the energy quantity and essential nutrients available to trigger or block different energy-demanding physiological process. The detection of nutrient levels by the central nervous system (CNS) is essential for the regulation of energy balance and, by extension, for the animal survival. Accordingly, neuronal circuits regulating food intake have developed mechanisms to detect changes in amino acid, fatty acid, and glucose levels that, in turn, have profound effects on their own activity to undertake nutritional demands (1, 2).

In vertebrates, glucose is one of the main nutrients whose changes in levels are sensed through different mechanisms (3). In mammals, the best known glucosensing system involves the phosphorylation of glucose to glucose 6-phosphate (G6P) by glucokinase (GK) that is then metabolized to increase ATP/ADP ratio that subsequently lead to the closure of ATP-dependent inward rectified potassium channels (K${}_{\text{ATP}}^{+}$) or Na^+^/K^+^_ATP_ (4). Chanel closure induces, in turn, membrane depolarization and the successive Ca^2+^ entry into the cell via L-type voltage-dependent calcium channel that results finally into increased neuronal activity [reviewed in (2, 5)]. In addition, alternative glucosensing mechanisms dependent on sodium/glucose cotransporter 1 (SGLT-1), liver X receptor (LXR), or sweet taste receptors has been proposed (2, 5). Neurons sensing glucose are classified in glucose-excited (GE) and glucose-inhibited (GI) based on their response to extracellular glucose levels by increasing or decreasing their firing rates, respectively. Therefore, the activation of glucosensing systems in the CNS regulates the neuronal activity and provokes regulatory responses allowing the animal to control glycemia via control the activity of the autonomic nervous system (6, 7). At central level, one of these responses involves the neural regulation of food intake. Thus, hypo- and hyperglycemia are known to increase and decrease food intake, respectively (7, 8). In fish, available evidence support the presence of comparable glucosensing mechanisms, either dependent or independent on GK [reviewed in (9, 10)]. These mechanisms are probably involved in the regulation of food intake and counter-regulatory mechanisms since hypo- and hyper-glycaemic treatments elicited also comparable changes in food intake (11, 12).

In mammalian species, brain glucose-sensing neurons are located in diverse areas, but especially in hypothalamus and brain stem (13). In the hypothalamus, glucose-sensing neurons are mainly found in the arcuate nucleus and ventromedial and lateral nuclei. In the brain stem, glucosensing cells are located in the area postrema, nucleus of the solitary tract and the dorsal motor nucleus of the vagus (7). In fact, hypoglycemia activates the above hypothalamic and caudal neurons as recorded by c-fos activity (14). There is also evidence supporting that glial cells, particularly astrocytes and tanycytes, located in the lower part of the third ventricle, are also involved in glucose sensing and may control the activity of hypothalamic neurons (5, 15–17). The nature of these glucosensing hypothalamic neurons is controversial (13, 18). Most studies have identified glusosensing neurons in different central areas but the phenotype remains elusive (13, 19). The arcuate nucleus incorporates two key neuronal populations involved in the control of food intake and feeding behavior. The first population co-expresses neuropeptide Y (NPY) and agouti-related peptide (AGRP), and the second population co-expresses pro-opiomelanocortin (POMC) and cocaine-and amphetamine-regulated transcript (CART) (1, 20, 21). Glucose regulates the activity of both populations (13) but the molecular mechanisms of glucosensing are still under debate.

Neural circuits and molecular mechanisms regulating feeding behavior are well-evolutionary conserved from fish to mammals (22). In fish, the melanocortin system is key in the regulation of feeding behavior (23–25). Thus, the intracerebroventricular injection of melanocortin agonists inhibits food intake in fasted goldfish (26). On the contrary, central administration of specific competitive antagonists of the melanocortin 4 receptor (MC4R) promotes feeding in satiated animals (26). Fasting has no effect on hypothalamic POMC expression but sharply stimulates the expression of the MC4R inverse agonist AGRP (27) that depress the activity of the constitutively expressed MC4R (28). In addition, the overexpression of inverse agonist of MC4R in transgenic models promotes feeding and growth in zebrafish (25). Prior experiments in fish using rainbow trout as a model have extensively demonstrated glucosensing capacity of central areas as well as the regulation of hypothalamic expression of neuropeptides by glucose availability through mechanisms comparable in general to those of mammals [reviewed in (10, 22)]. We have also described the presence of GK-immunoreactive cells in brain areas such as hypothalamus (29) and telencephalon (30) putatively involved in the control of food intake. However, there is no direct evidence to date supporting that neurons expressing GK are the same than those expressing the neuropeptides involved in the metabolic control of food intake. Accordingly, in this study, we have assessed, for the first time in fish, the simultaneous expression of marker of glucosensing such as GK in the melanocortinergic neurons expressing AGRP or POMC that actively regulate energy balance.

## Materials and Methods

### Fish

Female juvenile rainbow trout (*Oncorhynchus mykiss*, Walbaum 1792) ranging 80–100 g body weight were obtained from a local fish farm (A Estrada, Spain). Fish were maintained for 1 month in 100 liter tanks under laboratory conditions, 12L:12D photoperiod and dechlorinated tap water at 15°C. Fish weight was 98 ± 2 g. Fish were fed once daily (10:00 h) to satiety with commercial dry fish pellets (Dibaq-Diproteg SA, Spain; proximate food analysis was 48% crude protein, 14% carbohydrates, 25% crude fat, and 11.5% ash; 20.2 MJ/kg of feed). The experiments described comply with the Guidelines of the European Union Council (2010/63/UE), and of the Spanish Government (RD 53/2013) for the use of animals in research, and were approved by the Ethics Committee of the Universidade de Vigo.

### Synthesis of Riboprobes for ISH

Total RNA was extracted from rainbow trout hypothalamus using Trizol reagent (Life Technologies, Grand Island, NY, USA) and subsequently treated with RQ1-DNAse (Promega, Madison, WI, USA). A 2 μg sample of total RNA was reverse transcribed using Superscript II reverse transcriptase (Promega) and random hexamers (Promega) in approximately 20 μl volume. The cDNA obtained was subsequently used as template for PCR amplification with Taq DNA polymerase (Invitrogen) using specific primers based on AgRP1, GK, and POMCa1. The sequences were retrieved from GenBank sequence database (NCBI) with accession numbers NM001146677, AF053331, and NM001124718, respectively (Table 1). PCR fragments were separated onto 1% agarose gel, purified using EZNA Purification Kit (Omega Bio-tek), and subsequently were cloned into pGEM-T easy vector (Promega). Sense and antisense probes were synthesized using SP6/T7 RNA polymerase (Promega) using digoxigenin (DIG)-labeled UTPs or Fluorescein-labeled UTPs (Roche Diagnostic). The probes were treated with RQ1-DNAse-RNAse free (Promega) for 15 min at 37°C to remove the DNA template. Finally, the probes were purified using Micro Bio-Spin Chromatography Columns (BioRad) and quantified in a Thermo Scientific Nanodrop 2000c spectrophotometer.

**Table 1:** Primer sequences used for generate templates for synthesis of riboprobes for *in situ* hybridization.

	**Forward primer**	**Reverse primer**	**Size (bp)**	**GenBank accession number**
AGRP1	AGCAGTCCTGTCTGGGTAACACG	ATCCATCTACCCTATCGGGGAAAA	371	NM_001146677
GK	GCACGGCTGAGATGCTCTTTG	GCCTTGAACCCTTTGGTCCAG	170	AF053331
POMCa1	TCAGAAACCACTGCTCACG	GCTAACATCATCGCTATGTCAGAGGACAG	257	NM_001124718

*AgRP, Agouti-related peptide; GK, glucokinase; POMCa1, pro-opio melanocortin a1*.

### Tissue Preparation

Following 1 month acclimation period, 24-h fasted fish were anesthetized with 2-phenoxyethanol (Sigma, St. Louis, MO, USA; 0.2% v/v), perfused with physiological saline solution (NaCl 0.65%) and subsequently with the same volume of fixative containing paraformaldehyde (PAF, 4%) in phosphate buffer (PB, 0.1 M, pH 7.4). Freshly obtained tissues were removed, post-fixed overnight (O/N) in the same fixative at 4°C, dehydrated and embedded in Paraplast (Sigma). Transverse serial sections were cut at 7 μm using a rotary microtome and sections were mounted on 3-aminopropyltriethoxylane (TESPA)-treated slides and then air-dried at room temperature (RT) O/N.

### *In situ* Hybridization

Chromogenic ISH was used to detect the location of AgRP1 and POMC1a expression independently, and dual fluorescent *in situ* hybridization (FISH) was used on gene pairs (GK/AgRP1 and GK/POMC1a). Hybridizations with digoxigenin or fluorescein labeled sense or antisense riboprobes were revealed with either colorimetric 4-Nitro Blue Tetrazolium Chloride (NBT) and 5-Bromo-4-chloro-3-indoyl-phosphate (BCIP) staining (Roche Diagnostic), or fluorescent TSA (TSA PLUS Fluorescein kit, PerkinElmer) and Fast Red staining (Roche Diagnostic), respectively. ISH was performed as described by Agulleiro et al. (31), with some modifications as described below. Sections were deparaffinized, re-hydrated, post-fixed in PAF 4% for 20 min, and treated with Proteinase-K solution (20 μg/ml in 50 mM Tris-HCl, 5 mM EDTA at pH 8) for 8 min at RT. Slides were next washed in PB, post-fixed again in PAF for 5 min, subsequently rinsed in sterile water, and acetylated in a triethanolamine (0.1 M, pH 8)/acetic anhydride solution, before incubation with hybridization solution. Anti-sense or sense RNA probes were diluted in hybridization buffer containing 50% formamide, 300 mM NaCl, 20 mM Tris-HCl (pH 8), 5 mM EDTA (pH 8), 10% dextran sulfate (Sigma), and 1X Denhardt's solution (Sigma). Subsequently, 80–100 μl hybridization solution were added to each pretreated slide, which were coverslipped and incubated in a humidified chamber at 65°C O/N. Coverslips were removed by incubating slides into a solution containing 5X standard saline citrate buffer (SSC, contains 150 mM NaCl, 15 mM sodium citrate at pH 7) for 30 min at 55°C. The slides were then rinsed in 2X SSC and 50% formamide for 15 min at 65°C and three times immersed into NTE buffer (500 mM NaCl, 10 mM Tris-HCl, 5 mM EDTA, pH 7.5) for 5 min at 37°C. After ribonuclease treatment (40 μg/ml ribonuclease in NTE) for 15 min at 37°C, slides were rinsed in NTE buffer for 5 min at 37°C, once in 2X SSC and 50% formamide for 10 min at 65°C, once in 2X SSC for 10 min at RT and once in 0.1X SSC for 10 min at RT. Before incubated with antibody, slides were rinsed twice times in B1 (contains 100 mM Tris, 150 mM NaCl, pH 7.5) for 10 min at RT and washed with 2% blocking buffer (Roche Diagnostic) during 1 h. Slides were incubated at 4°C O/N with primary antibody 1:500 anti-digoxigenin FAB fragments conjugated to alkaline phosphatase (AP) antibody (Roche Diagnostic) in 2% blocking buffer. Redundant antibody was removed by washing twice in B2 (100 mM Tris, 100 mM NaCl, 50 mM MgCl_2_, pH 9.5) for 10 min at RT before incubated with chromogen substrates NBT/BCIP (Roche Diagnostic) to develop the staining. Sections were mounted with Mount quick acquoso (Bio-Optica) and visualized with Olympus BX41.

For dual fluorescence detection, slides were incubated O/N at 4°C in darkness with 1:100 anti-fluorescein FAB fragments conjugated to horseradish peroxidase (POD) antibody (Roche Diagnostic) in 2% blocking buffer. The next day, slides were washed twice in B1 with 0.1% triton for 10 min at RT, incubated for 20 min with tyramide-biotine (Fluorescein kit, PerkinElmer) in amplification diluent at RT, washed for 15 min at RT in Solution A with 0.1% triton (contains 100 mM Tris, 100 mM NaCl, pH 9.5), and incubated O/N at RT with Streptavidin (SA)—Alexa 488 (Life Technologies). Finally, slides were washed several times in PBS or PBS with 0.1% triton and subsequently developed with Fast Red staining or tyramide signal amplification (TSA) PLUS Fluorescein Kit. Sections were mounting with Prolong Gold antifade reagent with Dapi (Invitrogen) and visualized with Olympus BX41. Eight to ten sections of the target area (tuberal hypothalamus) from four different brains were used for double ISH.

## Results

**Figure 1:**
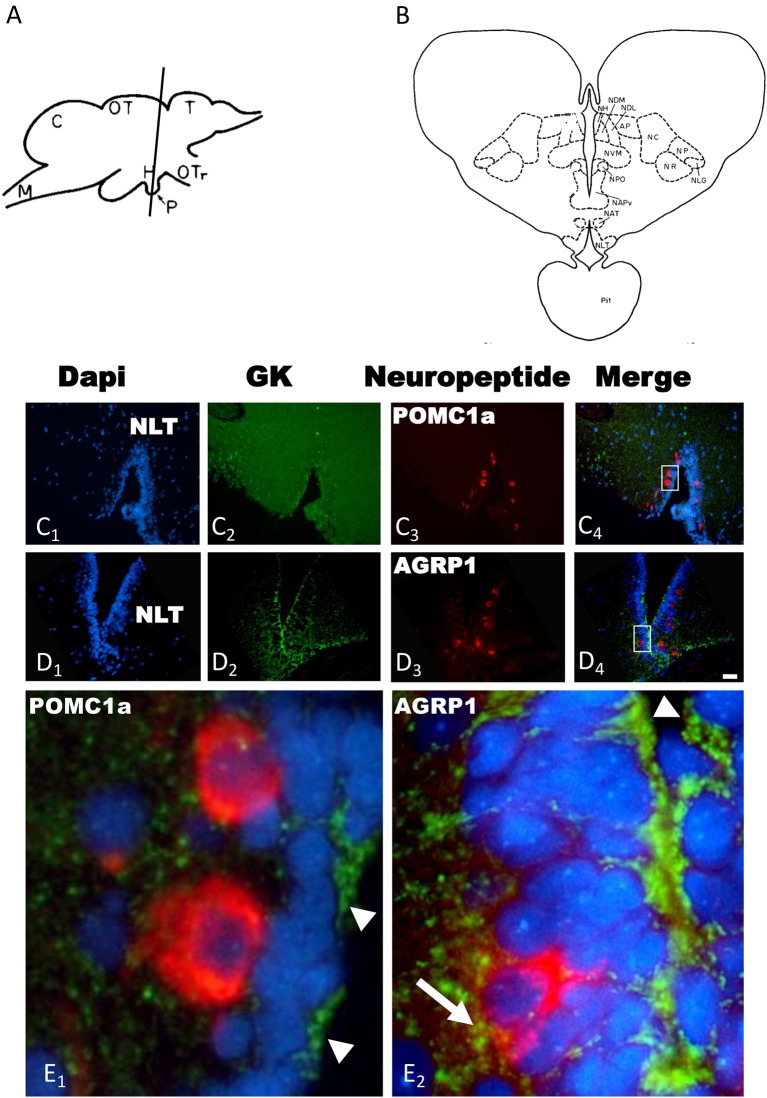
**(A)** Lateral view of the rainbow trout brain showing the levels of sections in **(C,D)** [adapted from Billard and Peter (32)]. **(B)** Schematic drawing of a transverse section showing the cytoarchitecture of the rostral trout hypothalamus at the level of **(C,D)** [adapted from Billard and Peter (32)]. Transverse sections of rainbow trout rostral hypothalamus showing GK **(C**_**2**_**,D**_**2**_**)**, POMC1a **(C**_**3**_**)** and AGRP1 **(D**_**3**_**)** mRNA expression by *in situ* hybridization. Slides were incubated with Dapi **(C**_**1**_**,D**_**1**_**)** to provide nuclear counterstaining of the same sections. Merge images is provided is **(C**_**4**_**,D**_**4**_**)**. Insets in **(C**_**4**_**,D**_**4**_**)** show magnification of GK/POMC1a **(E**_**1**_**)** or GK/AGRP1a **(E**_**2**_**)** colocalization. Arrowheads indicate GK mRNA expression in the ependymal cells **(E**_**1**_**,E**_**2**_**)**, arrow indicate potential coexpression of GK and AGRP1 mRNAs **(E**_**2**_**)**. AP, pretectal area; C, cerebellum; H, hypothalamus; M, medulla; NAPv, anterior periventricular nucleus; NAT, anterior tuberal nucleus; NC, cortical nucleus; NH, habenular nucleus; NDM, dorsomedial nucleus of the thalamus; NDL, dorsolateral nucleus of the thalamus; NLG, lateral geniculate nucleus; NLT, latertal tuberal nucleus; NP, pretectal nucleus; NPO, preoptic nucleus; NR, nucleus rotundus; NVM, ventromedial nucleus of the thalamus; OT, optic tectum; OTr, optic tract; Pit, pituitary; T, telencephalon. Scale bar 100 μm for **(C,D)** and 20 μm for **(E)**.

The *in situ* hybridization with the sense probes never generated specific signals in the trout brain (data not shown). Both POMC1a (Figure 1C_3_) and AGRP1 (Figure 1D_3_) localized exclusively in the rostral region of the ventral hypothalamus (Hv) also called ventral region of the lateral tuberal nucleus (NLTv). GK expression occurred in several brain regions such as hypothalamus, telencephalon, hindbrain, etc. Since neuropeptides localized exclusively in hypothalamus, we only described GK presence in hypothalamus. Thus, hypothalamic expression of GK was mostly associated to the ependymal cells that coat the wall of the third ventricle. GK-expressing ependymal cells localized mainly in the ventral region of the tuberal hypothalamus (Figures 1C_2_,D_2_). These cells imbricated between neurons coating the rostral region of the ventricle where both POMC and AGRP neurons are located. A higher magnification showed that GK did not express, or expressed at undetectable levels, in the hypothalamic POMC1a neurons (Figure 1E_1_). In contrast, hypothalamic AGRP1 neurons co-expressed GK (Figure 1E_2_).

## Discussion

Central glucosensing has been extensively studied in mammalian species and localized in key hypothalamic and caudal regions involved in the regulation of energy balance (6). However, the phenotypic characterization of central glucosensing neurons remains controversial and elusive (13). In fish, previous studies showed that hypothalamic mRNA abundance of POMC and AGRP respond to peripheral manipulation of systemic glucose (33–36). Furthermore, both types of neurons are involved in the regulation of energy balance in fish (25–27). Accordingly, hyper- or hypoglycaemic treatments inhibit or stimulate food intake in rainbow trout, respectively (11, 12). Our specific question was whether neurons that regulate the energy homeostasis, and particularly the melanocortinergic neurons, are able to sense glucose directly using GK system. To address this question, we evaluate the potential GK expression in both POMC and AGRP central neurons. Thanks to the low affinity for glucose, GK activity changes proportionally with glucose availability and it is thus considered to be a critical marker to predict glucosensing neuronal populations (6, 13) though not all glucosensing neurons express GK (13, 37).

POMCa1 and AGRP1 neurons localized exclusively in the ventral pole of the rostral lateral tuberal nucleus of the trout brain. The exclusive expression of POMC and AGRP in the ventral hypothalamus has been also reported in other fish species like goldfish (27), sea bass (38) and zebrafish (39). In the brain of tetrapods, AGRP expressed only in the neurons of the arcuate nucleus but POMC is expressed also in the caudal brain, particularly in the nucleus of the solitary tract (NTS) (40, 41). Based on the coincident phenotype of the neurons, the lateral tuberal nucleus has been proposed to be the homolog of the arcuate nucleus in fish. In mammals, a very high percentage of AGRP neurons co-express NPY (42) and regulate the activity of hypothalamic POMC neurons thanks to the γ-aminobutyric acid (GABA) projections (43, 44). Arcuate POMC neurons also produce CART but hypothalamus is not the only brain area producing this neuropeptide (45). Therefore, these antagonistic melanocortinergic populations, the orexigenic AGRP neurons and the anorexigenic POMC-expressing neurons, integrate metabolic, and nutrient information to regulate food intake. Recent experiments in fish suggest that the phenotype of AGRP neurons is a derived characteristic of mammalian brain since fish AGRP neurons does not produce NPY, however both zebrafish AGRP and NPY neurons are GABAergic and respond to fasting by increasing neuropeptide gene expression (46).

In mammals, orexigenic AGRP neurons are considered as GI neurons whereas POMC neurons seem to be GE neurons (18). In our hands, POMC1a neurons in the tuberal hypothalamus of rainbow trout do not exhibit GK-mediated glucosensing capacity since GK expression was under detection levels of the highly sensitive TSA technique (47).

Low expression levels of GK in the central nervous system has challenged the assessment of GK role in neuronal glucose sensing in mammalian species (48) and, according to the low level of GK expression detected in the trout hypothalamus, fish do not seem an exception. Therefore, data suggests that trout hypothalamic POMC neurons require presynaptic inputs to sense glucose. Obviously, trout POMC neurons respond to glucose since mRNA abundance of POMC usually increased in hypothalamus in response to a rise in glucose levels (33–36), but probably require presynaptic inputs from upstream neuronal cells. Rainbow trout were fasted for 24 h therefore we cannot rule out that fasting induced a decrease in plasma glucose that subsequently reduced differentially GK expression in POMC but no in AGRP neurons. However, short-term fasting in poikilotherms, including rainbow trout has no severe effects on glycemia (49). Studies using mice hypothalamic POMC dissociated neurons expressing GFP showed that glucose was unable to promote an increase in [Ca^+2^]i thus supporting the inefficiency of POMC neurons to directly detect changes in glucose levels (13). The glucose-induced modulation of frequency of excitatory postsynaptic currents onto POMC neurons also supports the requirements of presynaptic inputs of POMC neurons for sensing glucose (50).

How do POMC cells sense glucose then? One possibility would be that these cells respond to glucose through GK-independent mechanisms (2, 5). Another possibility, more likely, is that glucosensing might occur not in neurons alone but in a glucose-sensing unit including POMC neurons and glial cells as demonstrated in mammals (51, 52). Thus, according to our immunological data, we propose that ependymal cells, probably tanycytes, which coat the ventral pole of the third ventricle, send projections contacting with the closely located POMC neurons to convey information about glucose availability in the cerebrospinal fluid (CSF). In fact, tanycytes generate an interphase between CSF and neuronal nuclei that allows the exchange of molecules. Rat tanycytes express GLUT2 and GK and the functionality of the enzyme has been recorded thus suggesting the capability of tanycytes for glucose sensing (53). The adenovirus-mediated suppression of GK expression in the hypothalamic tanycytes after injection in the third ventricle increases feeding levels in rat but also depress hypothalamic POMC expression at the same time that preclude the AGRP neurons response to glucose administration (17). However, the expected response to glucose administration was to stimulate and inhibit POMC and AGRP expression, respectively (17). Our data suggest that trout hypothalamic AGRP1 neurons co-express GK indicating that they are potentially true glucosensing neurons based on this mechanism. To our knowledge, there is no data regarding GK expression on AGRP neurons but these orexigenic neurons respond to changes in glucose availability (17). Altered GK activity in the arcuate nucleus regulates the neuronal NPY secretion suggesting that GK modulates the activity of the NPY/AGRP neurons in the arcuate nucleus (54). In addition, GK expression is present in NPY arcuate neurons in rat (55). Since 95% of NPY neurons produce AGRP (42), it is conceivable that AGRP and GK co-expressed in the same neurons. GK co-expression in AGRP neurons and the putative real glucose sensing does not mean that the information coming from tanycytes about glucose was superfluous since suppression of GK in this ependymal cells also abolishes glucose response of AGRP neurons (see above).

In summary, our data support that POMC and AGRP neurons in rainbow trout brain are exclusively located in the ventral region of the lateral tuberal nucleus, i.e., the fish homolog to the mammalian arcuate nucleus. In this area, the main marker of glucosensing (GK) is expressed in the AGRP but not in POMC neurons. Therefore, we suggest that AGRP neurons respond directly to changes in glucose through GK-based glucosensing. In contrast, POMC neurons would not directly respond to glucose through GK and rather would require presynaptic inputs to sense glucose. Ependymal cells, particularly tanycytes coating the ventral region of the third ventricle, could play a key role in sensing CSF glucose and conveying nutrient information to central hypothalamic structures regulating energy balance, especially POMC neurons. Our results provide original information about specific hypothalamic nuclei in which nutrient sensors may interact with neuropeptides involved in the regulation of food intake.

## Ethics Statement

The experiments described comply with the Guidelines of the European Union Council (2010/63/UE), and of the Spanish Government (RD 53/2013) for the use of animals in research, and were approved by the Ethics Committee of the Universidade de Vigo.

## Author Contributions

JS and JC-R conceived and designed research. CO-R, RÁ-O, ES, and AR performed experiments. CO-R, RÁ-O, and JC-R analyzed data. CO-R, JS, and JC-R interpreted results. CO-R and JC-R prepared figures. All authors revised and edited the drafted manuscript. All authors approved the final version of manuscript.

### Conflict of Interest Statement

The authors declare that the research was conducted in the absence of any commercial or financial relationships that could be construed as a potential conflict of interest.
